# Effect of iron dose in maternal multiple micronutrient supplement on perceived side effects, adherence, acceptability and preferences: protocol for a randomised crossover trial

**DOI:** 10.1136/bmjopen-2026-118725

**Published:** 2026-05-27

**Authors:** Christopher R Sudfeld, Alfa Muhihi, Allison C Sylvetsky, Emmy Metta, Victoria S Brownlee, Erin M Oakley, Mohamed Bakari, Wafaie W. Fawzi, Shabani Kinyogoli, Jordan R Kuiper, Sabina Mugusi, Qing Pan, Mary M. Sando, Blair J. Wylie, Honorati Masanja, Emily R. Smith, Andrea B. Pembe

**Affiliations:** 1 Department of Global Health and Population, Harvard University T H Chan School of Public Health, Boston, Massachusetts, USA; 2 Africa Academy for Public Health, Dar es Salaam, Dar es Salaam, Tanzania, United Republic of; 3 Department of Nutrition, University of Rhode Island, Kingston, Rhode Island, USA; 4 Department of Behavioural Sciences, Muhimbili University of Health and Allied Sciences, Dar es Salaam, Dar es Salaam, Tanzania, United Republic of; 5 Department of Global Health, The George Washington University Milken Institute School of Public Health, Washington, District of Columbia, USA; 6 Department of Pediatrics and Child Health, Muhimbili University of Health and Allied Sciences, Dar es Salaam, Tanzania, United Republic of; 7 Department of Environmental and Occupational Health, The George Washington University Milken Institute School of Public Health, Washington, District of Columbia, USA; 8 Department of Clinical Pharmacology, Muhimbili University of Health and Allied Sciences, Dar es Salaam, Tanzania, United Republic of; 9 Department of Biostatistics & Bioinformatics, George Washington University, Washington, District of Columbia, USA; 10 Department of Obstetrics and Gynecology, Beth Israel Deaconess Medical Center, Boston, Massachusetts, USA; 11 Ifakara Health Institute, Dar es Salaam, Tanzania, United Republic of; 12 Department of Obstetrics and Gynecology, Muhimbili University of Health and Allied Sciences, Dar es Salaam, Tanzania, United Republic of

**Keywords:** NUTRITION & DIETETICS, OBSTETRICS, PUBLIC HEALTH, Africa South of the Sahara

## Abstract

**Introduction:**

Iron-folic acid (IFA) supplementation in pregnancy is recommended by the WHO, with a dose of 60 mg of iron in contexts where anaemia remains a severe public health problem. Iron-containing supplements may cause side effects that affect acceptability and adherence in a dose-response manner. Maternal multiple micronutrient supplements (MMS), which include iron and folic acid plus additional micronutrients, are also recommended in the context of rigorous research, and programmes are considering transitioning from IFA to MMS containing 30 mg of iron. We will evaluate the effect of iron dose in MMS on maternal acceptability, side effects, adherence and preferences.

**Methods and analysis:**

The Multiple Micronutrient Supplementation (MMS) Iron Dose Acceptability Crossover Trial is an individually randomised, quadruple-blind, non-inferiority crossover trial of daily antenatal MMS supplementation formulations that contain 60 mg, 45 mg and 30 mg elemental iron among pregnant women in Dar es Salaam, Tanzania. A total of 156 pregnant participants will be randomised to a sequence in which they receive each of the three MMS formulations for 1 month. Participants, investigators, outcome assessors and data analysts will be blinded to the treatment sequence. The primary trial outcome is participant-reported acceptability of each MMS formulation, measured on a Likert scale. Secondary and tertiary outcomes include preferred and least preferred formulation, identification of MMS formulation, reported side effects and adherence assessed by pill count. Regression analyses will be used to assess differences between formulations and will account for sequence and period effects of the crossover trial design. Qualitative in-depth interviews from a subsample of participants will be conducted to understand women’s perceptions and experiences taking the different MMS formulations.

**Ethics and dissemination:**

The trial protocol was approved by Harvard T. H. Chan School of Public Health Institutional Review Board (IRB), the Ifakara Health Institute IRB, the Muhimbili University of Health and Allied Sciences IRB, the National Health Research Ethics Sub-Committee and the Tanzania Medicine and Medical Device Authority. Results will be shared through publications and presentations at the local, regional and international levels.

**Trial registration number:**

ClinicalTrials.gov Identifier: NCT06069869.

**STRENGTHS AND LIMITATIONS OF THIS STUDY**

- Randomised design of the trial will provide causal evidence.
- The crossover design will enable the assessment of within-participant preferences and acceptability and will reduce between-person variability, thereby increasing statistical power.
- Qualitative data collection will contextualise and understand women’s experiences taking MMS with different iron doses
- Self-reported measures of acceptability may be prone to some degree of social desirability bias
- The trial will be conducted in Tanzania, and therefore, the findings, particularly on acceptability and preferences, may not be generalisable to all settings

## Introduction

 The WHO recommends daily iron-folic acid (IFA) supplementation for pregnant women, containing 30–60 mg of elemental iron, to prevent maternal anaemia, with 60 mg of iron being preferred in contexts where anaemia is a severe public health problem (>40% prevalence).^1^ Despite being recommended and implemented for decades, effective coverage of IFA supplementation in antenatal care (ANC) programmes (often measured by self-report of taking over 90 days of supplements during pregnancy) remains suboptimal in many countries.^2^ The reasons for low coverage identified in implementation research are multifactorial, including inconsistent IFA supply at ANC, missed ANC visits, inadequate counselling and communication from health providers, lack of perceived benefits, difficulty taking daily tablets, forgetfulness and side effects.^3^,^6^ Reported side effects of high-dose iron-containing supplements, such as nausea, heartburn, vomiting, constipation, diarrhoea, headache, dizziness, abdominal pain and fatigue, are non-specific and overlap with common symptoms experienced in pregnancy.^7 8^ The US Institute of Medicine established 45 mg/day of iron as the tolerable upper intake level during pregnancy (across all sources, including diet and supplement intake) based on potential gastrointestinal side effects, including nausea and constipation.^8^ The tolerable upper level refers to the maximum average daily intake of a nutrient that is unlikely to cause adverse health effects in nearly all individuals in the general population; the risk of side effects increases above the upper level. However, the most recent Cochrane Review, which included randomised trials comparing iron-containing supplements with placebo or control (many using 60 mg iron), found no difference in rates of nausea, heartburn or constipation and reported a lower risk of diarrhoea among women receiving IFA supplementation.^9^ As a result, the extent to which iron-containing supplements, particularly at higher doses, can cause side effects in pregnancy and the degree to which these may affect acceptability and adherence remains unclear.

Maternal multiple micronutrient supplements (MMS), or multivitamins that include iron and folic acid plus additional micronutrients, have been shown to reduce the risk of low birth weight and other adverse perinatal outcomes as compared with IFA supplements alone.^10 11^ MMS for pregnant women is currently recommended by the WHO in the context of rigorous research.^12^ The standard MMS formulation is the United Nations International Multiple Micronutrient Antenatal Preparation (UNIMMAP), which contains 15 vitamins and minerals, including 30 mg of elemental iron. Therefore, in settings with a high burden of anaemia where IFA supplements containing 60 mg of iron are currently used, it has been suggested that switching to MMS with 30 mg of iron may reduce perceived side effects and potentially improve acceptability and adherence. Nevertheless, studies have suggested that MMS and IFA, regardless of iron dose, have comparable rates of side effects and similar adherence.^13^ Anaemia in pregnancy remains a severe public health problem in Tanzania, affecting about 56% of pregnant women according to the 2022 Demographic and Health Survey.^14^ IFA supplements containing 60 mg of iron are the current standard of care for pregnant women in Tanzania, and the country is considering a transition to MMS containing 30 mg of iron. To the best of our knowledge, no randomised trials have directly compared MMS containing different iron dosages.

We will conduct a randomised crossover trial to evaluate supplement acceptability, side effects, adherence and preferences for MMS formulations containing 30, 45 or 60 mg of iron among pregnant women in Tanzania. The trial will include quantitative and qualitative assessments to comprehensively characterise participant acceptability and side effects, and evaluate factors underlying preferences and adherence patterns.

## Methods and analysis

The Multiple Micronutrient Supplementation (MMS) Iron Dose Acceptability Crossover Trial (MID-ACT) is an individually randomised, quadruple-blind, non-inferiority crossover trial of daily MMS supplements containing 60 mg, 45 mg or 30 mg of elemental iron among pregnant women in Dar es Salaam, Tanzania (clinicaltrials.gov registration: NCT06069869). The crossover design, in which each participant serves as their own control, was selected to allow for evaluation of within-participant preferences and acceptability, as well as reduce between-person variability to improve statistical power. We will also assess whether or not participants can identify the MMS formulations with higher iron dosages. Trial recruitment started on 26 September 2025 and was completed on 23 January 2026. The follow-up of participants is ongoing and is expected to be completed in late June 2026.

### Study Setting

Anaemia in pregnancy remains a severe public health problem in Tanzania.^14^ In line with WHO recommendations, IFA containing 60 mg of iron is currently provided to pregnant women as standard of care. Tanzania is currently considering switching to MMS with the 30 mg iron UNIMMAP formulation as the standard of care. The crossover trial will be conducted at the public antenatal care clinic at Kimara Health Centre in Dar es Salaam. The study clinic registers approximately 150 women per month for antenatal care and will therefore recruit the required sample size within 6 months. The clinic currently provides daily IFA containing 60 mg of iron as standard of care for pregnant women.

### Eligibility criteria and recruitment

The MID-ACT inclusion criteria are (1) pregnant women attending their first ANC visit at the study clinic, (2) ≤15 weeks of gestation based on the last menstrual period, (3) adult ≥18 years, (4) intending to stay in the study area for the duration of the study and (5) willing and able to provide written informed consent. Participants will be excluded if they meet any of the following criteria: (1) severe anaemia, defined as a haemoglobin concentration <8.5 g/dL per Tanzania standard of care; (2) presence of sickle cell disease (Hb-SS and Hb-SC) or haemoglobin C disease (Hb-CC); (3) concurrent participation in another nutritional supplementation trial and/or (4) a disability or condition that may impair the ability to provide informed consent and complete study procedures. At a screening visit, trained research staff will assess eligibility criteria for all individuals presenting to the antenatal care clinic and ask potential participants for written informed consent for trial enrolment. Following consent, a finger-prick blood sample will be collected to measure haemoglobin concentration using the HemoCue Hb 301 system (HemoCue AB, Ängelholm, Sweden). Sickle cell disease screening will be performed using the HemoTypeSC test (Silver Lake Research Corporation, Irwindale, USA). All participants will provide written informed consent to participate in the trial (online supplemental matrial).

### Interventions

Pregnant women enrolled in MID-ACT will each receive three different daily MMS formulations that contain 15 micronutrients but differ in iron dose. The formulations include the following: (1) MMS with 30 mg elemental iron (standard UNIMMAP formulation), (2) MMS with 45 mg elemental iron plus the standard UNIMMAP formulation for other nutrients and (3) MMS with 60 mg elemental iron plus the standard UNIMMAP formulation for other nutrients. The only difference between the MMS formulations will be the dosage of iron; all 14 other micronutrients will be at the same dosages. Iron will be provided in the supplements as ferrous sulfate. The trial MMS will be manufactured by DSM Nutritional Products South Africa (Gauteng, South Africa), and each will have an identical appearance, colour, odour, taste, size and weight. Therefore, the trial will evaluate the biological effect of the different iron dosages and not the difference in any physical property between the supplements. The MMS will be uniformly packaged in blister packs that contain a total of 35 tablets. Participants will take each of the three MMS formulations for 1 month (28 days).

### Randomisation and blinding

Pregnant women will be randomly assigned to the order in which they receive the three MMS formulations in the crossover trial. Pregnant women will be randomised in a 1:1:1:1:1:1 ratio to one of six treatment sequence orders to receive the three MMS formulations first, second or third in order (eg, 30, 45, then 60 mg; or 45, 30, then 60 mg; or 45, 60, then 30 mg, etc.) as shown in table 1. The allocation sequence will be generated by two non-study staff at George Washington University through computer-generated randomisation lists that will be block randomised (block sizes of 6). An independent study pharmacist will privately prepare a set of three MMS blister packs with participant identification numbers (IDs) according to the allocated treatment sequence. At the randomisation visit, research staff will assign pregnant women to the next available participant ID, which corresponds to a set of three prelabelled blister packs. Participants will receive an MMS blister pack at baseline, month 1 and month 2 visits. No washout period will be included between crossover periods. These randomisation procedures will ensure treatment allocation is fully concealed. Furthermore, the trial is quadruple-blind since the participants, investigators, outcome assessors and data analysts will be blinded to the participant treatment sequence.

**Table 1:** Multiple micronutrient supplementation treatment sequences in the crossover design

Regimen sequence	Month 1	Month 2	Month 3
Sequence 1	30 mg	45 mg	60 mg
Sequence 2	30 mg	60 mg	45 mg
Sequence 3	45 mg	30 mg	60 mg
Sequence 4	45 mg	60 mg	30 mg
Sequence 5	60 mg	30 mg	45 mg
Sequence 6	60 mg	45 mg	30 mg

### Data collection

**Figure 1:**
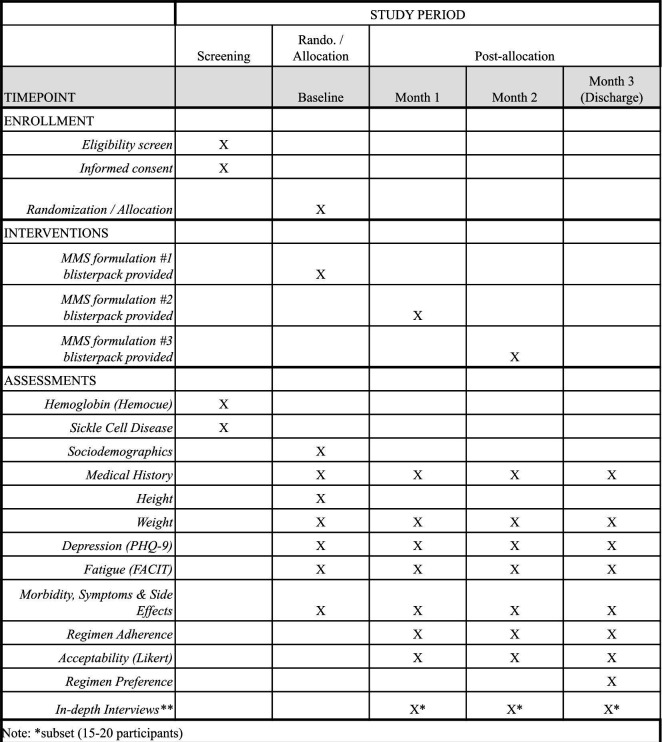
Schedule of enrolment, interventions and assessments in MID-ACT. MID-ACT, Multiple Micronutrient Supplementation (MMS) Iron Dose Acceptability Crossover Trial, FACIT, Functional Assessment of Chonic Illness Therapy - Fatigue Scale

Participants will have a baseline, 1-month follow-up, 2-month visit and 3-month visit (discharge). The schedule of assessments by visit is presented in figure 1. At baseline, participants will have sociodemographic characteristics assessed. Height will be measured by a stadiometer at the baseline visit. Maternal weight and mid-upper arm circumference will be taken at each study visit. At baseline and during pregnancy follow-up visits, participants will be seen by a study nurse, who will ask about their medical history and receive a clinical examination. Participants will be asked to recall morbidities, symptoms and side effects in the last 28 days at all visits. The Patient Health Questionnaire-9 and Functional Assessment of Chronic Illness Therapy (FACIT)-Fatigue Scale will be used to assess depression and fatigue symptoms, respectively, at all study visits.^15 16^ At each follow-up visit, study staff will take a pill count of tablets returned in MMS blister packs to assess adherence and participants will be asked about the acceptability of the MMS formulation they took in the last month, the primary trial outcome, using a continuous Likert scale ranging from 1 (disliked a lot) to 5 (liked a lot) to capture general impressions.

At the month 2 and month 3 follow-up visits, participants will be asked to compare the formulation taken during the current month with the MMS taken in the preceding month and to indicate any perceived differences or preferences. At the month 3 discharge visit, participants will be asked to reflect on all three MMS formulations and to report whether they perceived any differences among them, as well as whether they preferred or disliked any formulation relative to the others. Participants will also be asked to guess the order in which they received the MMS formulations by iron dose (eg, 30 mg first, 60 mg second, 45 mg third).

We will conduct in-depth interviews (IDIs) from a subsample of pregnant women in the MID-ACT crossover trial to understand women’s perceptions and experiences taking different formulations of MMS and the perceived acceptability, facilitators, barriers and side effects of antenatal iron supplements over time and across dosages. The sample size for the qualitative component will be approximately 20 participants; the exact sample size will be determined based on reaching saturation defined as when no new codes or themes emerge during qualitative analysis.^17^ Participants will be randomly selected from the MID-ACT trial cohort and invited to participate in the qualitative component of the trial; those who agree to participate will provide an additional written informed consent for the qualitative component. Each qualitative substudy participant will be scheduled to complete three IDIs: one at the month 1, month 2 and month 3 visits. Repeated IDIs will be conducted with the same participants at three timepoints to understand how women’s experiences and perceptions pertaining to taking MMS may differ over time and across the three iron dosages. Each IDI will be approximately 30–45 min in duration and will be conducted using a semi-structured IDI guide. The guide will be informed by the Theoretical Framework of Acceptability and include questions about women’s attitudes towards taking MMS, the perceived burden of MMS use, the perceived effectiveness of MMS, perceived benefits and side effects associated with MMS use and women’s confidence and willingness to take the supplements for the duration of their pregnancy.^18^ The questions will focus specifically on the MMS formulation taken within the past month, and additional questions about women’s experiences and preferences across dosages will be asked during the month 2 and month 3 visits.

IDIs will be transcribed verbatim and translated into English. During transcription, the transcriber will incorporate nonverbal cues observed during the interview. A second research team member will review each transcript against the audio recording to ensure accuracy and completeness. The translated transcripts will then be reviewed by a native Swahili speaker fluent in English to verify linguistic accuracy, clarity and preservation of the original meaning.

We will code all of the transcripts from the month 1 IDIs, prior to coding the transcripts from the month 2 and month 3 IDIs. We will use a multistep, iterative, open coding process. Two trained coders will review the transcripts to familiarise themselves with the data, after which, they will independently code an initial subset of the transcripts (n=5) in Microsoft Excel using a combined deductive and inductive approach: initial codes will consist of topics in the interview guide informed by the Theoretical Acceptability Framework (deductive) and additional topics and concepts that may emerge from the data. The two coders will meet to discuss the codes generated and create a shared codebook, which will be used to code the transcripts from the remaining month 1 IDIs. Additional inductive codes will be added as they emerge, and codes will be iteratively refined by merging and reorganising, as appropriate, to develop the final codebook. The two coders will then complete the final coding of all of the month 1 IDI transcripts using Dedoose. A subset of the transcripts (25%) will be coded by both coders to ensure consistency, and any discrepancies will be discussed among the coders and with a third research team member, if needed. After coding of all month 1 IDI transcripts is completed, the codes will be reviewed by a third research team member. Themes and subthemes will be identified using reflexive thematic analysis, after which, representative quotations will be selected. The month 2 and month 3 IDI transcripts will then be coded by the two coders using an analogous approach. Themes and subthemes identified across the month 1, 2 and 3 IDIs will then be compared to understand how women’s perceptions of and experiences with MMS may change across iron dosages and over time. The Tanzania and US qualitative research teams will collaborate throughout all stages of qualitative analysis, including codebook development, code interpretation, theme identification and manuscript writing.

### Data management

The study will use an electronic data capture system, with selected forms, including the informed consent form, maintained in paper format. Participants will be assigned unique study IDs, and direct identifiers will be stored separately from study data. All electronic data, including IDI audio recordings and transcripts, will use participant IDs and will be stored on password-protected, encrypted servers. Paper records will be stored in locked cabinets. All quantitative and qualitative analyses will be conducted using de-identified data, and no individual participants will be identifiable in reports or publications. These procedures were designed to ensure participant confidentiality.

In terms of data management procedures, the data system will include internal data quality checks, including automatic range checks, to identify data that appears to be inconsistent, incomplete or inaccurate. In addition to these real-time checks, the study data team will conduct routine data quality reviews. Data queries will be generated on a weekly basis to identify missing data, outliers and potential discrepancies. Queries will be issued to site staff for verification and resolution, with all changes tracked through an audit trail. Data quality metrics will be monitored throughout the study, and feedback will be provided to study sites as needed to maintain high data quality. Final data cleaning and validation will be completed before the database lock. The deidentified dataset supporting this research may be made available following a reasonable request submitted to the study team and completion of relevant ethical approvals and data transfer agreements.

### Concomitant care

All participants will be provided with antenatal care according to the national guidelines in Tanzania. Any woman who is identified as having severe anaemia (haemoglobin concentration <8.5 g/dL) during the study will be discontinued from taking study supplements and referred for treatment. The study team will obtain clinical trial insurance to ensure that participants who experience harm related to trial participation receive appropriate medical care. The study team will obtain clinical trial insurance to ensure that participants who experience harm related to trial participation receive appropriate medical care during or after the study. Unblinding of a participant’s allocated intervention will be permissible when necessary for participant safety, including requests related to medical care, and by the IRB.

### Outcomes

The primary, secondary and tertiary quantitative outcomes are presented in table 2. The primary outcome of the trial will be participant-reported acceptability of each MMS formulation using Likert scale scores ranging from 1 (disliked a lot) to 5 (liked a lot). Secondary outcomes include the most preferred MMS formulation, the least preferred MMS formulation, correct identification of iron dosage, any side effects of iron-containing supplements (diarrhoea, heartburn, constipation, vomiting, nausea, leg cramps or lower back/pelvic pain) and regimen adherence. Tertiary outcomes include participant-reported diarrhoea, heartburn, constipation, vomiting, nausea, leg cramps and lower back/pelvic pain.

**Table 2:** Primary, secondary and tertiary outcomes for MID-ACT

Primary outcome	Definition
Acceptability	Participant reported acceptability of each MMS formulation using Likert scale scores ranging from 1 (disliked a lot) to 5 (liked a lot).
**Secondary outcomes**	**Definition**
Most preferred MMS formulation	Participant-reported supplement formulation that was liked the best.
Least preferred MMS formulation	Participant-reported supplement formulation that was liked the least.
Identification of iron dosage	Percentage of participants who correctly identify their MMS iron dose randomised treatment order
Any side effect of iron	The percentage of patients experiencing any side effect (diarrhoea, heartburn, constipation, vomiting, nausea, leg cramps, lower back/pelvic pain) during each intervention period.
Adherence	The percentage of days a participant takes an MMS tablet (by pill count) among the total days in the intervention period
**Tertiary outcomes**	**Definition**
Diarrhoea	Self-reported diarrhoea during each intervention period
Heartburn	Self-reported heartburn during each intervention period
Constipation	Self-reported constipation during each intervention period
Vomiting	Self-reported vomiting during each intervention period
Nausea	Self-reported nausea during each intervention period
Leg cramps	Self-reported leg cramp during each intervention period
Lower back/pelvic pain	Self-reported lower back/pelvic pain during each intervention period

MID-ACT, Multiple Micronutrient Supplementation (MMS) Iron Dose Acceptability Crossover Trial; MMS, multiple micronutrient supplements.

### Sample size

This study is powered for the acceptability (positive impression per Likert scale) of MMS using a non-inferiority design. An empirical estimate of the SD is 0.76 based on real data from the non-inferiority of low-dose compared with standard high-dose calcium supplementation in pregnancy trial.^19^ Therefore, allowing for a non-inferiority margin of 0.5 point difference, 90% power and an alpha of 0.025 to accommodate two statistical tests for 60 versus 30 mg MMS and 45 versus 30 mg MMS (0.05/2), we would require a total of 147 women. To account for ~5% of missing or incomplete data (fetal loss, withdrawal etc.), we will therefore recruit 26 women for each of the six treatment sequences. Thus, the total sample size will be 156 participants.

### Statistical analysis

All primary quantitative analyses will be conducted using an intention-to-treat approach and limited to participants with observed outcome data (complete case). For the primary acceptability outcome, we will test two hypotheses: the mean Likert scale score in the 60 mg arm is non-inferior to the mean Likert scale score in the 30 mg arm; second, the mean Likert scale score in the 45 mg arm is non-inferior to the mean Likert scale score in the 30 mg arm. We will treat the Likert scale score as approximately continuous and use a linear mixed effects model for the analysis. A time-varying MMS formulation treatment indicator will be included as a covariate (treatment), and repeated monthly Likert scale scores from the same woman will share the same random intercept. To account for the crossover design, covariates for the randomised treatment sequence (sequence effect) and the visit month (period effect) will be included in the model.^20^ Two-sided 97.5% CI (to account for multiple comparisons) of the treatment effects will be presented and compared with the non-inferiority margin of −0.5 points. If most responses in the Likert scale concentrate in a few categories, cumulative link mixed models will be used.

Preference between two doses (45 mg vs 30 mg, and 60 mg vs 30 mg) will be analysed using the win ratio method to determine the preferred dose among each dose pair based on the preference of each participant. We collected the participants’ preferences for MMS formulations with 30 mg, 45 mg or 60 mg of iron by asking which supplement they liked the most or the least after taking all three MMS formulations. Take the 30 mg vs 60 mg comparison as an example. Logistic regression will be employed to estimate the probability that the 30 mg regimen is the preferred one among the two regimens for a specific woman, adjusting for differences in the periods of the 30 mg regimen and the 60 mg regimen, as well as the sequence of the three regimens (six sequences in total using five binary indicators). The sample size will be the number of pregnancies that received both the 30 mg and the 60 mg formulation. We will report the point estimate and 95% CIs for the percentage of participants favouring the 30 mg method as the winner. If the preference is not different between the 60 mg and the 30 mg formulations, the percentage of pregnancies choosing the 30 mg regimen as the winner should equal 50%. Similar analyses will be performed to compare 45 mg versus 30 mg.

Percent adherence will be modelled as a continuous outcome with a similar approach to the continuous Likert score outcome. A linear mixed effects model will be used with a time-varying covariate for MMS formulation (treatment), covariates for the sequence effect and period effect with random effects to account for repeated measures for each participant. For binary side effect outcomes (any side effect in the past month and individual symptom side effects reported in the past month), we will use log-binomial generalised estimating equation (GEE) models with MMS formulation as a time-varying covariate with covariates for sequence and period effects. Repeated measures within participants will be accounted for using the GEE model. We will assess differences in the number of days during the prior month that any side effect, as well as individual symptoms, was reported using GEE models with a log link and Poisson distribution. MMS treatment will be included as a time-varying exposure, with covariates for sequence and period effects, and will account for repeated measures. We will consider the adjustment of gestational age at the study visit and baseline side effects to potentially reduce extraneous variation and increase power.

## Ethics and dissemination

The MID-ACT protocol was approved by Harvard T. H. Chan School of Public Health Institutional Review Board (IRB) (Ref. No. IRB22-1581), the Ifakara Health Institute IRB (IHI/IRB/No.: 45–2023), the Muhimbili University of Health and Allied Sciences IRB (MUHAS-REC-07–2023-1801), the National Health Research Ethics Committee (Ref. No. NIMR/HQ/R.8a/Vol. IX/4448), Tanzania Commission for Science and Technology (Permit No. CST00000303-2024–2024-00342) and the Tanzania Medicine and Medical Device Authority (Ref No. BC.69/96/98/01). The George Washington University and Beth Israel Deaconess Medical Center IRBs relied on the Harvard IRB. All participants will provide written informed consent before enrolment into the trial and will have the right to withdraw at any time. All protocol modifications will be submitted to the trial IRBs for approval prior to implementation. Updates will also be made to the clinical trial registry, and approved amendments will be communicated to the study staff and to participants if relevant to participation or safety. An independent, external study monitor will conduct ongoing trial monitoring to ensure protocol compliance and data integrity.

In terms of dissemination, we will share the primary and secondary findings of our trial through publications in peer-reviewed journals and presentations at conferences, research seminars, and scientific meetings. We will work with the Healthy Mothers Healthy Babies Program hosted by the Micronutrient Forum to consider any other public-facing dissemination activities. Additionally, we will communicate our results to relevant government and non-governmental stakeholders, including the Tanzania Ministry of Health and Social Welfare and the Tanzania Food and Nutrition Centre, through the distribution of publications and an in-person dissemination meeting.

## Discussion

Our trial will provide causal evidence on maternal acceptability, side effects, adherence, and preferences, as well as robust qualitative data to contextualise and understand women’s experiences taking MMS with different iron doses. The trial findings are intended to inform public health programmes that are switching from higher to lower dose iron-containing supplements in pregnancy. In addition, we are conducting an ongoing efficacy trial to assess the clinical effect of MMS containing 30 mg, 45 mg and 60 mg on maternal anaemia and important perinatal outcomes (clinicaltrials.gov Identifier: NCT06079918).^21^ We will also conduct a crossover trial that will compare IFA containing 60 mg of iron with MMS containing 30 mg of iron to assess the total effect of the difference in iron dose and additional micronutrients on maternal acceptability (clinicaltrials.gov Identifier: NCT06069856). Taken together, these trials will provide robust evidence to inform decision-making regarding the transition from IFA to MMS in settings with a high anaemia burden.

## Supplementary material

10.1136/bmjopen-2026-118725 online supplemental file 1

## References

[1] World Health Organization (2016). WHO recommendations on antenatal care for a positive pregnancy experience.

[2] Sanghvi TG, Harvey PWJ, Wainwright E (2010). Maternal iron-folic acid supplementation programs: evidence of impact and implementation. Food Nutr Bull.

[3] Siekmans K, Roche M, Kung’u JK (2018). Barriers and enablers for iron folic acid (IFA) supplementation in pregnant women. Matern Child Nutr.

[4] Stoltzfus RJ (2011). Iron interventions for women and children in low-income countries. J Nutr.

[5] Nisar YB, Dibley MJ, Mir AM (2014). Factors associated with non-use of antenatal iron and folic acid supplements among Pakistani women: a cross sectional household survey. BMC Pregnancy Childbirth.

[6] Kebaabetswe P, Diseko M, Zash R (2024). A qualitative assessment of barriers to iron and folic acid supplementation among pregnant women in Botswana. BMC Public Health.

[7] Tolkien Z, Stecher L, Mander AP (2015). Ferrous sulfate supplementation causes significant gastrointestinal side-effects in adults: a systematic review and meta-analysis. PLoS ONE.

[8] Institute of Medicine (2001). Dietary Reference Intakes for Vitamin A, Vitamin K, Arsenic, Boron, Chromium, Copper, Iodine, Iron, Manganese, Molybdenum, Nickel, Silicon, Vanadium, and Zinc.

[9] Finkelstein JL, Cuthbert A, Weeks J (2024). Daily oral iron supplementation during pregnancy. Cochrane Database Syst Rev.

[10] Keats EC, Haider BA, Tam E (2019). Multiple-micronutrient supplementation for women during pregnancy. Cochrane Database Syst Rev.

[11] Smith ER, Shankar AH, Wu L-F (2017). Modifiers of the effect of maternal multiple micronutrient supplementation on stillbirth, birth outcomes, and infant mortality: a meta-analysis of individual patient data from 17 randomised trials in low-income and middle-income countries. Lancet Glob Health.

[12] World Health Organization (2020). Nutritional interventions update: multiple micronutrient supplements during pregnancy.

[13] Kissell MC, Pereira C, Gomes F (2025). Acceptability of Antenatal Multiple Micronutrient Supplementation (MMS) Compared to Iron and Folic Acid (IFA) Supplementation in Pregnant Individuals: A Narrative Review. Nutrients.

[14] Ministry of Health (2023). Tanzania demographic and health survey and malaria indicator survey 2022 key indicators report.

[15] Kroenke K, Spitzer RL, Williams JB (2001). The PHQ-9: validity of a brief depression severity measure. J Gen Intern Med.

[16] Yellen SB, Cella DF, Webster K (1997). Measuring fatigue and other anemia-related symptoms with the Functional Assessment of Cancer Therapy (FACT) measurement system. J Pain Symptom Manage.

[17] Saunders B, Sim J, Kingstone T (2018). Saturation in qualitative research: exploring its conceptualization and operationalization. Qual Quant.

[18] Sekhon M, Cartwright M, Francis JJ (2017). Acceptability of healthcare interventions: an overview of reviews and development of a theoretical framework. BMC Health Serv Res.

[19] Dwarkanath P, Muhihi A, Sudfeld CR (2021). Non-inferiority of low-dose compared to standard high-dose calcium supplementation in pregnancy: study protocol for two randomized, parallel group, non-inferiority trials in India and Tanzania. Trials.

[20] Lim C-Y, In J (2021). Considerations for crossover design in clinical study. Korean J Anesthesiol.

[21] Smith ER, Muhihi A, Wylie BJ (2025). Multiple micronutrient supplementation for maternal anemia prevention (MMS-MAP): an individually randomized trial of higher-dose iron (60 mg, 45 mg) compared to low-dose iron (30 mg) in multiple micronutrient supplements in pregnancy. Trials.

