# Supplementary material for: Effect of iron dose in maternal multiple micronutrient supplement on perceived side effects, adherence, acceptability and preferences: protocol for a randomised crossover trial
Source: BMJ Open. 2026 May 27;16(5):e118725. doi: 10.1136/bmjopen-2026-118725 (PMC13218192; doi:10.1136/bmjopen-2026-118725)
Supplement: online supplemental file 1 [file bmjopen-16-5-s001.pdf]

Online Supplemental Material

This document includes the Multiple Micronutrient Supplementation (MMS) Iron Dose Acceptability Crossover Trial (MID-ACT) participant consent forms for (1) trial enrollment and (2) qualitative interviews.

Participant ID:__________________

Protocol Title: Randomized trial of higher-dose iron (60 mg, 45 mg) compared to low dose iron (30 mg) in multiple micronutrient supplements in pregnancy on moderate and severe maternal anemia – ***MMS Iron Dose Acceptability Crossover Trial***
Principal Investigators: Prof. Andreas Pembe (MUHAS), Dr. Honorati Masanja (IHI), Dr. Alfa Muhihi (AAPH), Dr. Blair Wylie (Beth Israel Deaconess Medical Center), Dr. Christopher Sudfeld (Harvard) and Dr. Emily Smith (GWU)
**Description of Study Population:** 156 Pregnant women in Tanzania
**Version Date:** March 2, 2025

MMS IRON DOSE ACCEPTABILITY CROSSOVER TRIAL CONSENT

Greetings! My name is _________________________ and I am a nurse working on a research project. The research is trying to see whether giving different doses of iron in multivitamin supplements to pregnant women has an impact on side effects and adherence. The study is being done jointly by Muhimbili University of Health and Allied Sciences (MUHAS), Ifakara Health Institute (IHI), Africa Academy for Public Health (AAPH) in Tanzania and the Harvard T.H. Chan School of Public Health, Beth Israel Deaconess Medical Center, and the George Washington University School of Public Health (GWU) in the U.S. The aim of this discussion is to inform you about this research activity and to ask for your consent to participate in the study.

Key Information

The following is a short summary of this study to help you decide whether or not to participate. More detailed information is listed later on in this form.

Why am I being invited to take part in a research study?

We have invited you to take part in a research study because you are a pregnant woman under 15 weeks pregnant who is not severely anemic or has sickle cell disease.

What should I know about a research study?

- Someone will explain this research study to you.
- Whether or not you take part is up to you.
- You can choose not to take part.
- You can agree to take part and later change your mind.
- Your decision will not be held against you.
- You may discuss your decision with your family, your friends and/or your doctor.
- You can ask all the questions you want before you decide.

Why is this research being done?

We are conducting research to determine the best dose of iron in multivitamins for pregnant women in terms of side effects and adherence.

Participant ID:___________________

How long will I take part in this research?

You will take part in this study for 4 months while you are pregnant.

What will I be asked to do?

In this study, you will take multivitamins for 3 months but each month we will give you multivitamins with a different dose of iron in a random but specific order. You will be randomized to take multivitamins that contain 30 mg, 45 mg, or 60 mg of iron in a specific order. For example, some pregnant women will receive the low dose 30 mg multivitamins first, high dose multivitamins with 60 mg iron second, and medium dose multivitamins with 45 mg third; while others will randomly receive the high iron dose first, medium dose second, and low dose third and other people will receive different orders. We would then ask you to attend monthly clinic visits during your participation in this study– three visits in total. We would ask you about your adherence, any side effects, and health, have a doctor examine you, and assess your nutritional status at each visit while you are pregnant. At the exit visit after 4 months, we will also ask you to tell us what you thought about the multivitamins and which you liked and which you did not like. We will also invite some women to participate in an in-depth interview on multivitamins in the future. We will contact you if you are selected to potentially participate in an in-depth interview. After the study, you will continue to receive IFA supplements at the ANC clinic.

More detailed information about the study procedures can be found under the *"What can I expect if I take part in this research*?" section.

Is there any way being in this study could be bad for me?

Taking multivitamins that contain iron during pregnancy lowers the risk of maternal anemia. However, some pregnant women may have mild side effects from taking multivitamins with iron including nausea, dizziness, abdominal discomfort, diarrhea, constipation, and headaches. There is also a small risk of breach of confidentiality.

More detailed information about the risks of this study can be found under the *"What are the risks and possible discomforts?"* section.

Will being in this study help me in any way?

Multivitamins containing iron will be provided for free to all pregnant women. This study will help us find out if there is the best iron dose in multivitamins for adherence and side effects. We hope that the information we learn from this study may benefit other pregnant women. We do not expect that any additional costs to you will result from taking part in this study. There are no plans to give you money for taking part in this study.

What happens if I do not want to be in this research?

Participation in research is voluntary. You can decide to participate, not to participate, or stop participation at any time without penalty or loss of benefits to which you are otherwise entitled. Instead of being in this research study, your choice is not to participate.

Participant ID:__________________

Detailed Information

To follow, please find more detailed information about this study than already provided above.

About this consent form

Please read this form carefully. This form provides important information about participating in research. You have the right to take your time in making decisions about participating in this research. You may discuss your decision with your family, your friends and/or your doctor. If you have any questions about the research or any portion of this form, please ask us. If you decide to participate in this research, you will be asked to sign this form. A copy of the signed form will be provided to you for your record.

Participation is voluntary

You are invited to take part in this research because you are pregnant under 15 weeks gestation and are not severely anemic or have sickle cell disease. It is your choice whether or not to participate. If you choose to participate, you may change your mind and leave the study at any time. Refusal to participate or stopping your participation will involve no penalty or loss of benefits to which you are otherwise entitled.

What you should know about a research study

- Someone will explain this research study to you.
- A research study is something you volunteer for.
- Whether or not you take part is up to you.
- You can choose not to take part in the research study.
- You can agree to take part now and later change your mind.
- Whatever you decide it will not be held against you.
- Feel free to ask all the questions you want before you decide.

What is the purpose of this research?

The purpose of this research is to try and see if there is a difference in adherence and side effects for multivitamins with different doses of iron during pregnancy. Iron and folic acid supplementation are currently recommended by the Government of Tanzania. Multivitamins, which contain iron and folic acid plus additional vitamins and nutrients, have been shown to improve infant birthweight as compared to iron and folic acid supplementation. Multivitamins are currently not recommended for all pregnant women by the Government of Tanzania. There are some concerns about the iron content of multivitamins and this study will help determine the best dose of iron to include in multivitamins for pregnant women.

How many people will take part in this research?

Participant ID:__________________

About 156 pregnant women will take part in this research.

How long will I take part in this research?

You will be enrolled in the study for 4 months. There is a chance that you may be selected and invited for an in-depth interview. We will contact you to discuss this if you are selected, and there will be a separate consent for that.

What can I expect if I take part in this research?

First visit: When you join the study, we will randomize you to the order you receive multivitamins that contain 30, 45, or 60 MMS. This means you will receive multivitamins for all 3 months but will have an equal chance of which multivitamin dose you take first, second or third, like the flip of a coin. We are conducting this study because we are not sure what dose of iron is best for adherence and side effects. As a result, we do not know if the 30 mg iron, 45 mg iron or 60 mg iron multivitamins will be better for you, or if all three groups are equal. Neither you, your nurse, or any researcher involved in doing the study will be able to know which order you take the multivitamins until the research is finished.

At your first visit, we will ask you questions about your health, your household, occupation, and education. You may skip any study questions or procedures that you are not comfortable answering, and it will not affect your medical care. We will also give you blister packs of study multivitamin supplements and will ask you to take one tablet per day during pregnancy. If you consent, we will also ask you about the location of your home. It is helpful to us to find out where you live so that we can visit you at home sometimes to follow up on your health, at a time that is convenient for you. This first visit will take about 45 minutes to complete in addition to your routine clinic visit time.

Clinic Visit at Month 1, Month 2 and Month 3: We will ask you to come to clinic visits once a month for 3 months. At each of these visits, you will be asked questions about your health since the last visit as well as about your adherence and side effects of the multivitamins. We will also measure your nutritional status including weight, height, and arm size at each visit. These follow-up visits during pregnancy will take about 30 minutes or 45 minutes. If you have moved since your last visit, we will ask your permission to accompany you to your new address so that we can record it. If you miss any of these study visits, we would like permission to contact you to find out how you are doing. At the end of the last visit, at 4 months, we will also ask you questions on which multivitamins you like and which you do not like.

What are the risks and possible discomforts?

Taking multivitamins that contain iron during pregnancy lowers the risk of maternal anemia. However, we do not know the best dose of iron is best for adherence and side effects. As a result, this is why we are conducting this study. Some pregnant women may have mild side effects of taking multivitamins with iron including nausea, dizziness, abdominal discomfort, diarrhea, constipation, and headaches. There is also a small risk of breach of confidentiality. We will not share your health information with anyone who is not part of the study.

Are there any benefits from being in this research study?

Multivitamins containing iron will be provided for free to all pregnant women. We are not sure whether taking multivitamins with 60 mg, 45 mg, or 30 mg of iron per day is best for adherence. This study will help us find out if one of the multivitamins is more beneficial for adherence and side effects.

Participant ID:__________________

We hope that the information we learn from this study may benefit other pregnant women. We do not expect that any additional costs to you will result from taking part in this study. There are no plans to give you money for taking part in this study.

What are my alternatives to participating in this research?

The alternative to participating in this research study is not to participate.

Can I still get medical care at this health clinic if I choose not to participate in this research?

Yes, you may still get medical care if you choose not to participate in this study. Your decision will not change the care you receive now or in the future. Taking part in this research is your choice. If you decide to take part in this study, you may leave/stop the study at any time. There will be no penalty to you and your medical care will not be affected. If you would like to stop participating in this research, you should let us know. We will make sure that you stop the study safely. It is possible that the investigator may ask you to stop the study before it is finished. If this happens, we will tell you why and arrange for other care for you if needed.

Will I be compensated for participating in this research?

We will provide you compensation for transportation to the clinic for extra visits that are not included in the standard ANC schedule and as a result, we will compensate you 2,000 TZ Shillings for each extra visit.

What will I have to pay for if I participate in this research?

It will not cost you anything to participate in this research.

What happens if I am injured as a result of participating in this research study?

If physical injury resulting from participation in this research should occur to you, although we will not provide compensation, medical treatment will be available including first aid, emergency treatment, and follow-up care as needed. In making such medical treatment available, or providing it, the persons conducting this research project are not admitting that your injury was their fault.

Can my taking part in the research end early?

You may decide not to continue your participation in the research at any time without it being held against you. The person in charge of the research can remove you from the research at any time without your approval for any reason. Possible reasons for removal include if you start taking medicines or have a diagnosis that makes it unsafe for you to take multivitamins or it is not in your best interest to continue the study.

If I take part in this research, how will my privacy be protected? What happens to the information you collect?

We will assign a study identification number to you. All information we collect on forms will be entered into computers under this study identification number. All interactions regarding your health will take place in private cubicles. All your medical information will be stored in a locked room here and only

Participant ID:__________________

people who work for the study will have access to this information. Data collected, including your identifiable information, may be seen by the IRB that oversees the research.

If I have any questions, concerns or complaints about this research study, who can I talk to?

The Tanzania overall Principal Investigator of this study: Prof. Andrea Pembe at Muhimbili University of Health and Allied Sciences (MUHAS), Dar es Salaam, Phone: +XXXXXXXXXX

- If you have questions, concerns, or complaints,
- If you would like to talk to the research team,
- If you think the research has hurt you, or
- If you wish to withdraw from the study.

This research has been reviewed by the Institutional Review Boards of Muhimbili University of Health and Allied Sciences, Ifakara Health Institute and the National Institute for Medical Research (NIMR).

- If you wish to speak with someone else at MUHAS, you can call the office of the director of research and publications +XXXXXXXXXX
- If you wish to speak with someone at IHI, you can call +XXXXXXXXXX. IHI office is located on plot # 463, Kiko avenue, Mikocheni, Dar es Salaam.
- If you wish to speak with someone from the NIMR you can call +XXXXXXXXXX. NIMR is located at 2448 Ocean Road P.O. Box 9653 in Dar es Salaam.

Statement of Consent

I have read the information in this consent form including risks and possible benefits. All my questions about the research have been answered to my satisfaction. I understand that I am free to withdraw my participation at any time without penalty.

1. In case you move out of Dar-es-Salaam during the study period, do you agree that a research assistant can contact your neighbors or relatives to learn about your health?

Participant agrees__________________ Participant does NOT agree______________

2. Do you consent to a home visit if you miss a clinic visit? Only experienced trackers will visit your home if you consent and they will not disclose that they are medical staff or part of a study team. They will also wear plain clothes and questions asked of your neighbors and family will be limited to your whereabouts.

Participant agrees________________ Participant does NOT agree______________

3. Do you agree to allow us to store your information to use in later research studies in pregnancy on condition that ethical institutional review boards (committees) in Tanzania and in the United States approve of such requests?

Participant agrees________________ Participant does NOT agree______________

Participant ID:__________________

4. Do you agree to allow us to share your information with other investigators for use in later research studies on pregnancy?

Participant agrees______________ Participant does NOT agree________________

SIGNATURE

Name of Participant ____

Signature of Participant____

Signature of Legally Authorized Representative____
(When applicable)

Signature of Research Assistant ____

Date of signed consent __________________________

If illiterate

I have witnessed the accurate reading of the consent form to the potential participant, and the individual has had the opportunity to ask questions. I confirm that the individual has given consent freely.

*Print name of witness*_______________________ *AND* *Thumb print of participant*

Signature of witness ________________________

Date __________________________

Participant ID:__________________

**Protocol Title:** Randomized trial of higher-dose iron (60 mg, 45 mg) compared to low dose iron (30 mg) in multiple micronutrient supplements in pregnancy on moderate and severe maternal anemia – ***MMS Iron Dose Acceptability Crossover Trial: In-depth Interview***	
Principal Investigators: Prof. Andreas Pembe (MUHAS), Dr. Honorati Masanja (IHI), Dr. Alfa Muhihi (AAPH), Dr. Blair Wylie (Beth Israel Deaconess Medical Center), Dr. Christopher Sudfeld (Harvard) and Dr. Emily Smith (GWU)	
**Description of Study Population:** 15-20 Pregnant women in Tanzania	
**Version Date:** March 2, 2025	

Greetings! My name is __________________________ and I am a nurse working on a research project. The research is trying to see whether giving different doses of iron in multivitamin supplements to pregnant women affects side effects and adherence. The study is being done jointly by Muhimbili University of Health and Allied Sciences (MUHAS), Ifakara Health Institute (IHI), Africa Academy for Public Health (AAPH) in Tanzania and the Harvard T.H. Chan School of Public Health, Beth Israel Deaconess Medical Center, and the George Washington University School of Public Health (GWU) in the U.S. The aim of this discussion is to inform you about this research activity and to ask for your consent to participate in the study.

Key Information

The following is a short summary of this study to help you decide whether or not to participate.

About this consent form

Please read this form carefully. This form provides important information about participating in research. You have the right to take your time in making decisions about participating in this research. You may discuss your decision with your family, your friends and/or your doctor. If you have any questions about the research or any portion of this form, please ask us. If you decide to participate in this research, you will be asked to sign this form. A copy of the signed form will be provided to you for your record.

Why am I being invited to take part in a research study?

We have invited you to take part in a research study because you participated in the MMS Iron dose crossover study, and that you have been randomly selected to participate in in-depth interviews.

Participation is voluntary

It is your choice whether or not to participate. If you choose to participate, you may change your mind and leave the study at any time. Refusal to participate or stopping your participation will involve no penalty or loss of benefits to which you are otherwise entitled.

What should I know about a research study?

- Someone will explain this research study to you.
- Whether or not you take part is up to you.
- You can choose not to take part.
- You can agree to take part and later change your mind.

Participant ID:___________________

- Your decision will not be held against you.
- You may discuss your decision with your family, your friends and/or your doctor.
- You can ask all the questions you want before you decide.

Why is this research being done?

We are conducting research to determine the best dose of iron in multivitamins for pregnant women in terms of side effects and adherence. We will hold in-depth interviews to hear about pregnant women's experiences with the multivitamins they received while participating in the study.

How many people will take part in this research?

About 15-20 people will take part in this research.

How long will I take part in this research?

You will be enrolled in the study for the duration of three in-depth interviews which occur once a month for three months.

What will I be asked to do?

You will be asked to individually participate in three in-depth interviews with study staff. These in-depth interviews will take place after each monthly follow-up visit. We will electronically record the audio (no video) of the in-depth interview. This in-depth interview will take about 1.5 hours to complete.

Is there any way being in this study could be bad for me?

There are no foreseeable risks to participation. In addition, there is a small risk of breach of confidentiality of your statements in the in-depth interview recordings.

Will being in this study help me in any way?

There are no direct benefits to participating. We hope that the information we learn from this study provides a better understanding of side effects and adherence to different doses of iron in the multivitamin supplement in Tanzania and other settings.

What happens if I do not want to be in this research?

Participation in research is voluntary. You can decide to participate, not to participate, or stop participation at any time without penalty or loss of benefits to which you are otherwise entitled. Instead of being in this research study, your choice is not to participate.

What are my alternatives to participating in this research?

The alternative to participating in this research study is not to participate.

Can I still get medical care at this health clinic if I choose not to participate in this research?

Yes, you may still get medical care if you choose not to participate in this study. Your decision will not change the care you receive now or in the future. Taking part in this research is your choice. If you decide to take part in this study, you may leave/stop the study at any time. There will be no penalty to you and your medical care will not be affected. If you would like to stop participating in this research, you should let us know. We will make sure that you stop the study

Participant ID:___________________

safely. It is possible that the investigator may ask you to stop the study before it is finished. If this happens, we will tell you why and arrange for other care for you if needed.

Will I be compensated for participating in this research?
An allowance of 20,000 TZ Shillings will be provided for each in-depth interview. Payment will only be given for attending and participating in the in-depth interview.

What will I have to pay for if I participate in this research?
It will not cost you anything to participate in this research.

Can my taking part in the research end early?
You may decide not to continue your participation in the research at any time without it being held against you.

If I take part in this research, how will my privacy be protected? What happens to the information you collect?
We will assign a study identification number to you. All information we collect on forms will be entered into computers under this study identification number. The in-depth interviews will be audio recorded. Data collected, including your identifiable information, may be seen by the Harvard Institutional Review Board (IRB) and The National Institute for Medical Research (NIMR) that oversees the research.

If I have any questions, concerns or complaints about this research study, who can I talk to?

The Tanzania overall Principal Investigator of this study: Prof. Andrea Pembe at Muhimbili University of Health and Allied Sciences (MUHAS), Dar es Salaam, Phone: +XXXXXXXXXX

- If you have questions, concerns, or complaints,
- If you would like to talk to the research team,
- If you think the research has hurt you, or
- If you wish to withdraw from the study.

This research has been reviewed by the Institutional Review Boards of Muhimbili University of Health and Allied Sciences, Ifakara Health Institute and the National Institute for Medical Research (NIMR).

- If you wish to speak with someone else at MUHAS, you can call the office of the director of research and publications +XXXXXXXXXX
- If you wish to speak with someone at IHI, you can call +XXXXXXXXXX. IHI office is located on plot # 463, Kiko avenue, Mikocheni, Dar es Salaam.
- If you wish to speak with someone from the NIMR you can call +XXXXXXXXXX. NIMR is located at 2448 Ocean Road P.O. Box 9653 in Dar es Salaam.

Statement of Consent

I have read the information in this consent form including risks and possible benefits. All my questions about the research have been answered to my satisfaction. I understand that I am free to withdraw my participation at any time without penalty.

Participant ID:___________________

SIGNATURE

Name of Participant ____

Signature of Participant____

Signature of Legally Authorized Representative____
(When applicable)

Signature of Research Assistant ____

Date of signed consent _________________________

If illiterate

I have witnessed the accurate reading of the consent form to the potential participant, and the individual has had the opportunity to ask questions. I confirm that the individual has given consent freely.

*Print name of witness*______________________ *AND* *Thumb print of participant*

Signature of witness _______________________

Date _________________________
