# Supplementary material for: Effect of iron dose in maternal multiple micronutrient supplement on perceived side effects, adherence, acceptability and preferences: protocol for a randomised crossover trial
Source: BMJ Open. 2026 May 27;16(5):e118725. doi: 10.1136/bmjopen-2026-118725 (PMC13218192; doi:10.1136/bmjopen-2026-118725)
Supplement: Supplementary file 2 [file bmjopen-2026-118725.reviewer_comments.pdf]

PEER REVIEW HISTORY

BMJ Open publishes all reviews undertaken for accepted manuscripts. Reviewers are asked to complete a checklist review form and are provided with free text boxes to elaborate on their assessment. These free text comments are reproduced below.

ARTICLE DETAILS

Title (Provisional)

Effect of iron dose in maternal multiple micronutrient supplement on perceived side effects, adherence, acceptability, and preferences: Protocol for a randomised crossover trial

Authors

Sudfeld, Christopher R.; Muhihi, Alfa; Sylvetsky, Allison C; Metta, Emmy Mwita; Brownlee, Victoria S.; Oakley, Erin M.; Bakari, Mohamed; Fawzi, Wafaie.; Kinyogoli, Shabani; Kuiper, Jordan R.; Mugusi, Sabina; Pan, Qing; Mwanyika-Sando, Mary; Wylie, Blair; Masanja, Honorati; Smith, Emily; Pembe, Andrea B

VERSION 1 - REVIEW

**Reviewer**	**1**
**Name**	**Heslehurst, Nicola**
**Affiliation**	**Newcastle University**
**Date**	**23-Apr-2026**
**COI**	**None**

This manuscript reports the study protocol for MMS Iron Dose Acceptability Crossover Trial (MID-ACT) - a randomised crossover trial exploring the effect of iron dose in multiple micronutrient supplements in pregnancy in Tanzania, including perceived side effects, adherence, acceptability and preferences. This is an important and timely study to address maternal nutrition and pregnancy outcomes.

This is a well written protocol for an important study. I have some minor comments for amendments - subject to confirmation that the protocol is eligible for publication based on the stage of study (see comments on methods).

Introduction:

- The authors state the difference in doses recommended by WHO for contexts where anamia is a severe public health problem, and that the study is set in Tanzania. The prevalence of maternal anaemia in Tanzania is not mentioned until the methods/study setting (Anemia in pregnancy remains a severe public health problem in Tanzania, affecting

about 56% of pregnant women according to the 2022 Demographic and Health Survey [14]) and would be best placed in the introduction to introduce why Tanzania is the setting for the research before it is mentioned as the context (page 5 line 35).

Methods:

- there are no dates of the study included (as required by the journal) and no indication of whether this is a prospective protocol manuscript before any elements of the study have commenced, or a retrospective protocol submission and the study is already on-going (and if so, what stage is it at). The ISRCTN dates suggest that the study was registered originally in 2023 and is complete? Copied below from the registration.

Study Start (Actual)

2025-09-26

Primary Completion (Actual)

2026-01-23

Study Completion (Estimated)

2026-04-24

If the study is complete, the protocol would not be eligible for publication according to the journal requirements for the publication of protocols. If it is ongoing, then the authors need to provide dates in the manuscript and the current status - ensuring it adheres to the journal requirements.

- Page 9 line 27 - "The sample size for the qualitative component will be approximately 20 participants; the exact sample size will be determined based on reaching saturation." - how is saturation being determined? Data saturation, meaning saturation? Needs to be explicit what method will be used.

- Good description of the language translation for interviews to ensure English language retains meaning. Will the two coders include at least one researcher from Tanzania to ensure that the meaning and context is maintained during analysis?

Dissemination - how will results be communicated to government and non-government stakeholders, and who are these? Will there be an public facing dissemination activities?

- Patient and public involvement: Patients and the public were not involved in the trial design. - can the authors justify why the target population was not involved in the study design, especially considering that their perceived side effects, adherence,

acceptability, and preferences are key outcomes?

SPIRIT checklist has several items where the authors have stated N/A when it is not clear why as it appears that they are applicable?

- Recruitment 20 Strategies for achieving adequate participant enrollment to reach target sample size - why is this deemed N/A?

- 24c If blinded, circumstances under which unblinding is permissible, and procedure for revealing a participant's allocated intervention during the trial - why is this deemed N/A?

- Data management 26 Plans for data entry, coding, security, and storage, including any related processes to promote data quality (e.g., double data entry; range checks for data values). Reference to where details of data management procedures can be accessed, if not in the protocol - why is this deemed N/A?

- Monitoring - all marked as N?A - if there is no monitoring then 29 trial monitoring needs to have an explanation

- Protocol amendments 31 Plans for communicating important protocol modifications to relevant parties - why N/A?

- Confidentiality 33 How personal information about potential and enrolled participants will be collected, shared, and maintained in order to protect confidentiality before, during, and after the trial - why N/A?

- Ancillary and post-trial care 34 Provisions, if any, for ancillary and post-trial care, and for compensation to those who suffer harm from trial participation - why N/A?

VERSION 1 - AUTHOR RESPONSE

Reviewer #1

This manuscript reports the study protocol for MMS Iron Dose Acceptability Crossover Trial (MID-ACT) - a randomised crossover trial exploring the effect of iron dose in multiple micronutrient supplements in pregnancy in Tanzania, including perceived side effects, adherence, acceptability and preferences. This is an important and timely study to address maternal nutrition and pregnancy outcomes.

This is a well-written protocol for an important study. I have some minor comments for amendments - subject to confirmation that the protocol is eligible for publication based on the stage of study (see comments on methods).

Introduction:
- The authors state the difference in doses recommended by WHO for contexts where anamia is a severe public health problem, and that the study is set in Tanzania. The prevalence of maternal anaemia in Tanzania is not mentioned until the methods/study setting (Anemia in pregnancy remains a severe public health problem in Tanzania, affecting about 56% of pregnant women according to the 2022 Demographic and Health Survey [14]) and would be best placed in the introduction to introduce why Tanzania is the setting for the research before it is mentioned as the context (page 5 line 35).

Response: We agree that this is an important point to add to the introduction. We have added the following sentences to the second paragraph:

"Anemia in pregnancy remains a severe public health problem in Tanzania, affecting about 56% of pregnant women according to the 2022 Demographic and Health Survey [14]. IFA supplements containing 60 mg of iron are the current standard of care for pregnant women in Tanzania, and the country is considering a transition to MMS containing 30 mg of iron."

Methods:
- there are no dates of the study included (as required by the journal) and no indication of whether this is a prospective protocol manuscript before any elements of the study have commenced, or a retrospective protocol submission and the study is already on-going (and if so, what stage is it at). The ISRCTN dates suggest that the study was registered originally in 2023 and is complete? Copied below from the registration.
Study Start (Actual)
2025-09-26
Primary Completion (Actual)
2026-01-23
Study Completion (Estimated)
2026-04-24

If the study is complete, the protocol would not be eligible for publication according to the journal requirements for the publication of protocols. If it is ongoing, then the authors need to provide dates in the manuscript and the current status - ensuring it adheres to the journal requirements.

Response: Thank you for catching an error in the clinicaltrials.gov record. January 23, 2026, was the date that recruitment of participants was completed, not the completion of study follow-up. The correction to the anticipated date of primary completion has been posted to clinicaltrials.gov. The trial data collection is ongoing and will be completed by late June, as the final participant will exit the 60-day late visit window on June 24, 2026.

We have added the following language regarding the current status in the methods section:

"Trial recruitment started on September 26, 2025, and was completed on January 23, 2026. The follow-up of participants is ongoing and is expected to be completed by late June 2026."

- Page 9 line 27 - "The sample size for the qualitative component will be approximately 20 participants; the exact sample size will be determined based on reaching saturation." - How is

saturation being determined? Data saturation, meaning saturation? Needs to be explicit what method will be used.

Response: We have revised the manuscript to define how saturation will be determined in the qualitative component. Thematic/data saturation will be considered reached when no new codes or themes emerge during analysis, consistent with established qualitative research standards (Saunders et al., 2018).

"The sample size for the qualitative component will be approximately 20 participants; the exact sample size will be determined based on reaching saturation, defined as when no new codes or themes emerge during qualitative analysis (Saunders et al. 2018)."

- Good description of the language translation for interviews to ensure English language retains meaning. Will the two coders include at least one researcher from Tanzania to ensure that the meaning and context is maintained during analysis?

Response: The Tanzanian research team will be leading or engaged throughout all stages of qualitative analysis, including codebook development, interpretation of codes, and identification of themes and subthemes. This will help ensure that local context, meaning, and cultural nuance are appropriately reflected in the analysis and interpretation of findings.

We have added the following to the qualitative methods section: "The Tanzania and US qualitative research teams will collaborate throughout all stages of analysis, including codebook development, code interpretation, theme identification, and manuscript writing."

Dissemination - how will results be communicated to government and non-government stakeholders, and who are these? Will there be public-facing dissemination activities?

Response: We have revised the manuscript to specify that findings will be disseminated through the circulation of publications and an in-person dissemination event as well as public-facing activities. We will include several key stakeholders (e.g., the Tanzania Ministry of Health and Social Welfare and the Tanzania Food and Nutrition Centre). Additional stakeholder engagement will evolve based on interest in MMS implementation; we prefer not to provide a list of other potential government institutions and NGO stakeholders in the protocol.

We have added the following statement to the dissemination section:

"We will work with the Healthy Mothers Healthy Babies Program hosted by the Micronutrient Forum to consider any other public-facing dissemination activities. Additionally, we will communicate our results to relevant government and non-governmental stakeholders, including the Tanzania Ministry of Health and Social Welfare and the Tanzania Food and Nutrition Centre, through the distribution of publications and an in-person dissemination meeting."

- Patient and public involvement: Patients and the public were not involved in the trial design. - can the authors justify why the target population was not involved in the study design, especially considering that their perceived side effects, adherence, acceptability, and preferences are key outcomes?

Response: **We agree that patient and public involvement is important. Our study includes quantitative and qualitative components designed to evaluate patient-centered outcomes, with the primary outcome being participant-reported acceptability and secondary outcomes including preferences, perceived side effects, and adherence, all measured directly from patients. These approaches are intended to capture multiple aspects of acceptability and experience that patient involvement would typically inform. Therefore, given the nature of the trial design and research questions, the patients and the public were not directly included in the design.**

SPIRIT checklist has several items where the authors have stated N/A when it is not clear why as it appears that they are applicable?

Response: **We agree there were multiple places for better documentation on the SPIRIT Checklist. We have detailed the clarifications below.**

- Recruitment 20 Strategies for achieving adequate participant enrollment to reach target sample size - why is this deemed N/A?

Response: We have detailed the number of new ANC attendees per month at the study clinic

"The study clinic registers approximately 150 women per month for antenatal care and will therefore recruit the required sample size within 6 months."

- 24c If blinded, circumstances under which unblinding is permissible, and procedure for revealing a participant's allocated intervention during the trial - why is this deemed N/A?

Response: We have detailed the unblinding options related to participant safety in the *Concomitant care* section of the manuscript.

"Unblinding of a participant's allocated intervention will be permissible when necessary for participant safety, including requests related to medical care, and by the IRB."

- Data management Plans for data entry, coding, security, and storage, including any related processes to promote data quality (e.g., double data entry; range checks for data values). Reference to where details of data management procedures can be accessed, if not in the protocol - why is this deemed N/A?

Response: We have clarified the data management procedures in a new section of the methods on *Data Management*.

“In terms of data management procedures, the data system will include internal data quality checks, including automatic range checks, to identify data that appears to be inconsistent, incomplete, or inaccurate. In addition to these real-time checks, the study data team will conduct routine data quality reviews. Data queries will be generated on a weekly basis to identify missing data, outliers, and potential discrepancies. Queries will be issued to site staff for verification and resolution, with all changes tracked through an audit trail. Data quality metrics will be monitored throughout the study, and feedback will be provided to study sites as needed to maintain high data quality. Final data cleaning and validation will be completed prior to database lock.”

- Monitoring - all marked as N?A - if there is no monitoring then 29 trial monitoring needs to have an explanation

Response: We have clarified that the study has an external monitor.

“An independent, external study monitor will conduct ongoing trial monitoring to assess protocol compliance.”

- Protocol amendments 31 Plans for communicating important protocol modifications to relevant parties - why N/A?

Response: We have added details on the protocol amendment process to the IRB section of the manuscript.

“All protocol modifications will be submitted to the trial IRBs for approval prior to implementation. Updates will also be made to the clinical trial registry, and approved amendments will be communicated to the study staff and to participants if relevant to their participation or safety.”

- Confidentiality 33 How personal information about potential and enrolled participants will be collected, shared, and maintained in order to protect confidentiality before, during, and after the trial - why N/A?

Response: We have added procedures related to patient data and information to the *Data Management* section of the methods.

“The study will use an electronic data capture system, with selected forms, including the informed consent form, maintained in paper format. Participants will be assigned unique study IDs, and direct identifiers will be stored separately from study data. All electronic data, including IDI audio recordings and transcripts, will use participant IDs and will be stored on password-protected, encrypted servers. Paper records will be stored in locked cabinets. All quantitative and qualitative analyses will be conducted using de-identified data, and no individual participants will be identifiable in reports or publications. These procedures were designed to ensure participant confidentiality.”

- Ancillary and post-trial care 34 Provisions, if any, for ancillary and post-trial care, and for compensation to those who suffer harm from trial participation - why N/A?

Response: We have further detailed our requirement for clinical trial insurance in the *Concomitant care* section of the manuscript.

"The study team will obtain clinical trial insurance to ensure that participants who experience harm related to trial participation receive appropriate medical care during or after the study."
